# Supplementary material for: A double-spiral maze and hi-resolution tracking pipeline to study dispersal by groups of minute insects
Source: Sci Rep. 2023 Mar 30;13:5200. doi: 10.1038/s41598-023-31630-8 (PMC10063622; doi:10.1038/s41598-023-31630-8)
Supplement: Supplementary file 1 — Supplementary Information. [file 41598_2023_31630_MOESM1_ESM.pdf]

A double-spiral maze and hi-resolution tracking pipeline to study dispersal by groups of minute insects

Cointe, M.[a]*, Burte, V. [a], Perez, G. [a], Mailleret, L. [ab] & Calcagno, V. [a]

[a] Université Côte d'Azur, INRAE, CNRS, Institut Sophia Agrobiotech, Sophia Antipolis, France

[b] Université Côte d'Azur, Inria, INRAE, CNRS, Sorbonne Université, Biocore, Sophia Antipolis, France

*Author for correspondence: melina.cointe@inrae.fr

Supplementary Material

Material	Number of units	Unit price (€)	Total price (€)	Commercial reference
Opaque Plexiglas plate of 1000x1000 mm	1	34.4	34.4	Richardson
Transparent Plexiglas plate of 1000x1000 mm	2	40.95	40.95	Richardson
Camera Nikon D810	1	1298	1298	Idealo.fr
Use of a LASER cutting machine hourly rate	1	60	60	SoFab Sophia Antipolis
			1433.4	

Table S1. Evaluation of the cost of production and use of the double spiral maze. The dimensions of the sold plates actually allow the production of two double spiral mazes.

Strain	Test Duration (hours)	Mean number of individuals in the tested zones	Leaks observed by eye	Parasitized sentinel eggs	Tested zones
**PMBIO1**	48	78	None	0	17,16,11,19,31,27,37
**PMBIO1**	48	46	None	0	6,8,16,17,19,22,26,37
**ACJY144**	48	128	None	0	1,3,13,21,25,29,39
**PMBIO1**	48	71	None	0	10,2,35,28
**PJ**	48	60.5	None	0	5,15,7,30,36,20
**PUG029**	48	64.2	None	0	32,38,24,12,9,14
**PUG029**	48	73	None	0	17,16,11,19,27,34
**PMBIO1**	48	61	None	0	6,8,22,37,31
**ACJY144**	48	44.4	None	0	33,1,3,13,21,25,23,39
**PMBIO1**	48	65,2	None	0	10,2,35,28,15
**PMBIO1**	48	86	None	0	5,15,7,30,36,20
**PMBIO1**	48	76.5	None	0	32,38,24,12,9,14

Table S2. Results of the quality of sealing test

Strain	Introduced Individuals	Remaining individuals inside the tube	Total of individuals	Number of eggs on a strip introduced in the tube	Introduction Rate
PMBIO1	45	38	83	100	54.2
PMBIO1	78	51	129	150	60.5
ACJY144	99	60	159	220	62.3
PJ	109	45	154	600	70.8
PMBIO1	166	48	214	500	77.6
PMBIO1	96	60	156	200	61.5
PMBIO1	92	65	157	200	58.6
PMBIO1	63	27	90	200	70
PMBIO1	62	24	86	200	72.1
PMBIO1	92	70	162	200	56.8
PMBIO1	90	73	163	200	55.2
PMBIO1	80	40	120	200	66.7
PMBIO1	79	30	109	200	72.5

Table S3. Results of the introduction of individuals test

Strain	Sample Location	Capture Plant	Sampling year
ACJY144	Aime, France	Ash tree	2015
BL103b	Le Change	Primrose	2016
CAS052	Castellane	Boxwood	2016
Citron	Grasse	Lemon tree	2012
FLO239	Le Crouzet	Rose bush	2016

**GOT0130**	Gotheron	Apple tree	2016
**ISA1075**	Maurice de Beynost	Plum tree	2015
**MES008**	Beaumat	Maple tree	1987
**PJ**	France	Laboratory strain	2015
**PMBIO1**	France	Unknown	2014
**PUG233**	La Pugère	Apple tree	2015
**TCMz**	France	Unknown	1987
**TCVigne**	France	Unknown	1987
**TSM008**	Tours sur Meymont	Apple tree	2015

Table S4. Information on the 14 strains used in the results section: their name, their sampling location, the plant on which they were captured and their sampling year.

**Strain**	**Diffusion Coefficient_1**	**Diffusion Coefficient_2**	**Diffusion Coefficient_3**	**Breakpoint_1**	**Breakpoint_2**
**TCVigne**	36,8	85,95	44,33	45,84	255,88
**TSM008**	78,35	64,9	43,09	172,5	294,13
**CAS052**	28,89	71,53	37,53	31,09	217,89
**PUG233**	41,97	65,99	35,07	61,87	215,09
**GOT0130**	33,3	43	29,67	71,06	314,01
**MES008**	40,44	71,57	43	76,36	316,1
**TCMz**	54,97	100,59	35,13	90,16	227,1
**PJ**	12,31	24,89	34,29	106,12	292,51
**Citron**	29,66	69,37	None for type2	45,18	None for type2
**PMBIO1**	35,54	54,86	None for type 2	80,58	None for type 2
**Strain**	**Diffusion Coefficient**	**Constant**			
**BL103b**	49,49	149,87			

Table S5. Summary of diffusion coefficients and breakpoints for all remaining strains not shown in the main figures. “Citron” and “PMBIO1” represent the remaining type 2 strains, “BL103b” represents the remaining linear strain, and all the other strains represent type 3 strains.

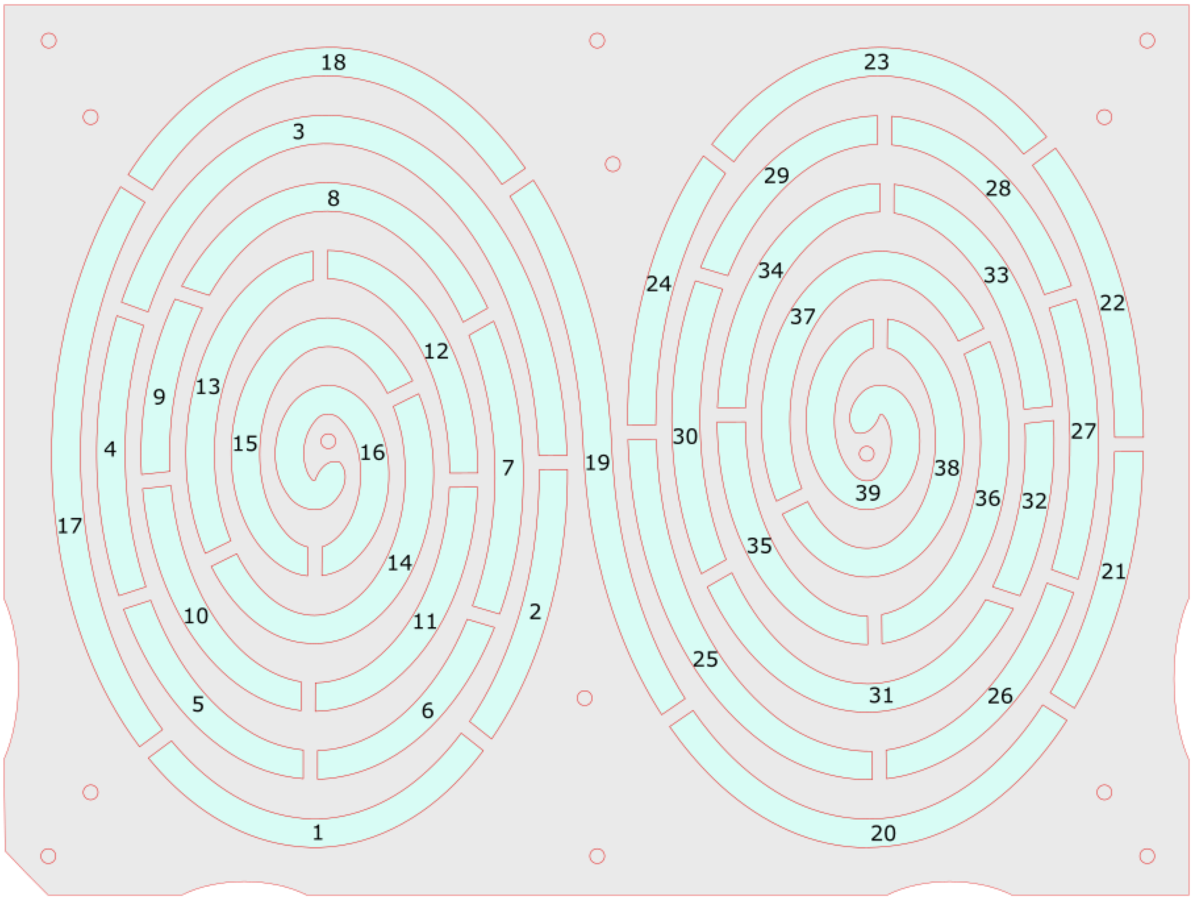

Figure S1. A variant of the (levogyrous) double-spiral maze that was engineered with solid walls (5mm width), or septa, along the tunnel, in order to test for the quality of sealing. This was designed with the Inkscape software, delineating 39 isolated zones (in light blue on the scheme). The numbers correspond to the zones called in table 2. An SVG file ready for laser-cutting is available as Supplementary File.

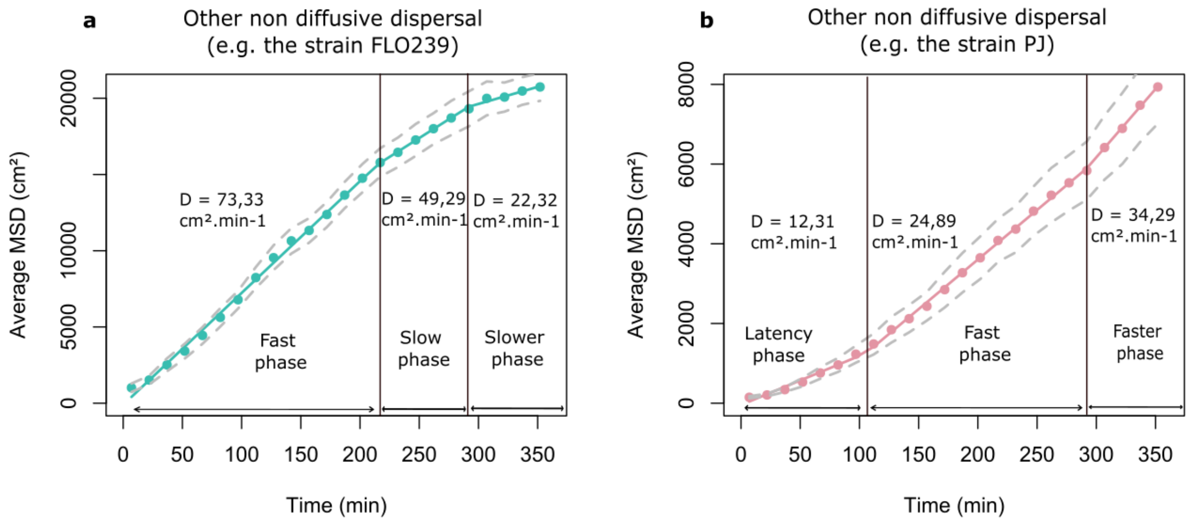

Figure S2. The two strains of the category "Others'' in figure 6E which have a non-diffusive dispersal as with three distinct phases as in type 3, but specific patterns: FLO239 (a) is characterized by a first fast phase and then two successive slow phases. PJ (b), on the contrary, is characterized by a latency phase and two successive fast phases. Remark: we used piecewise linear regressions to test for deviations from linearity, but in those cases, it is possible that changes in diffusion coefficients are gradual rather than discrete: i.e. panel (a) might represent a gradual deceleration of spread, whereas panel (b) may feature a gradual acceleration of spread.

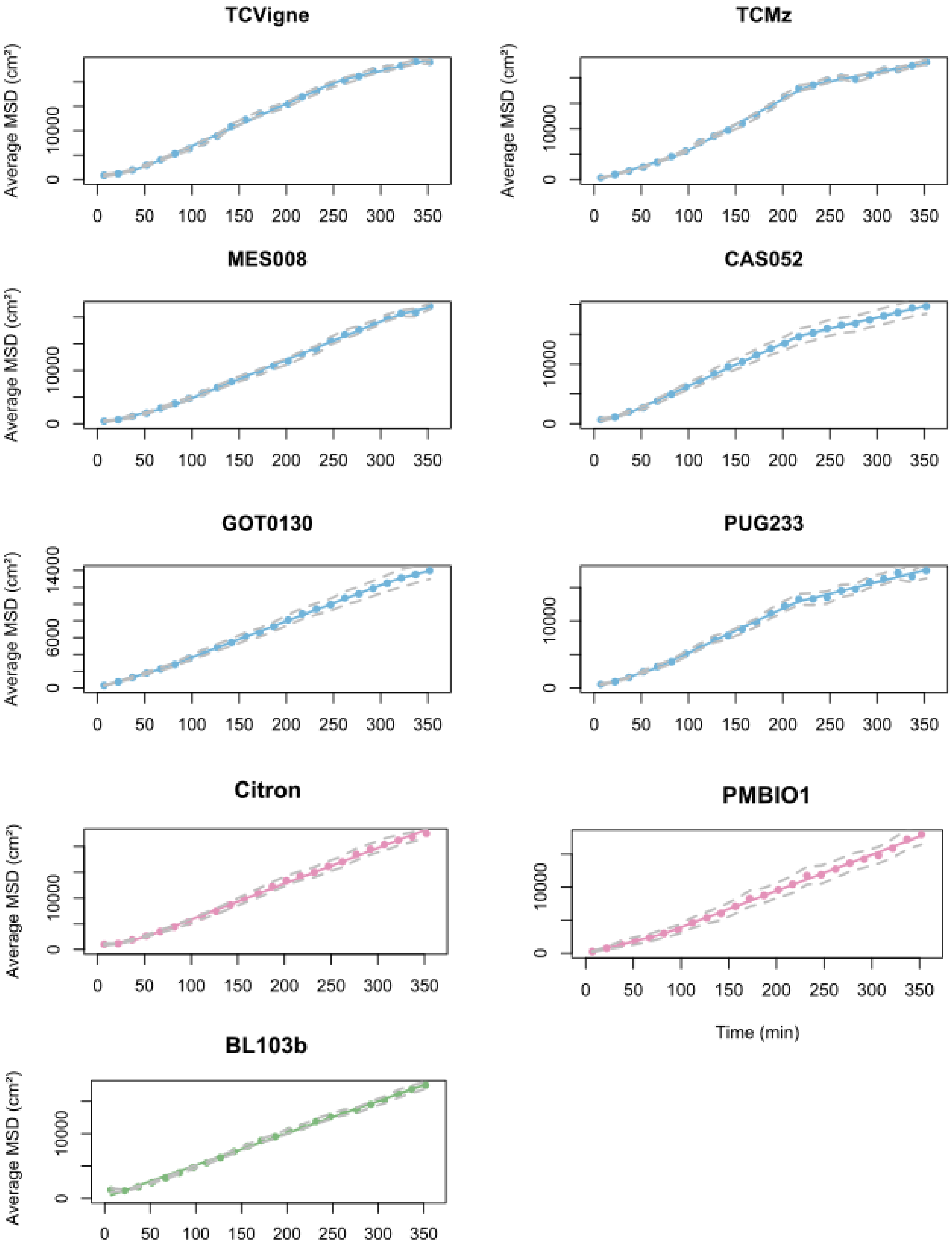

Figure S3. Plots representing the Mean Squared Displacement over time for the remaining studied strains. In blue the strains of type3, in pink the strains of type2, in green the linear type.

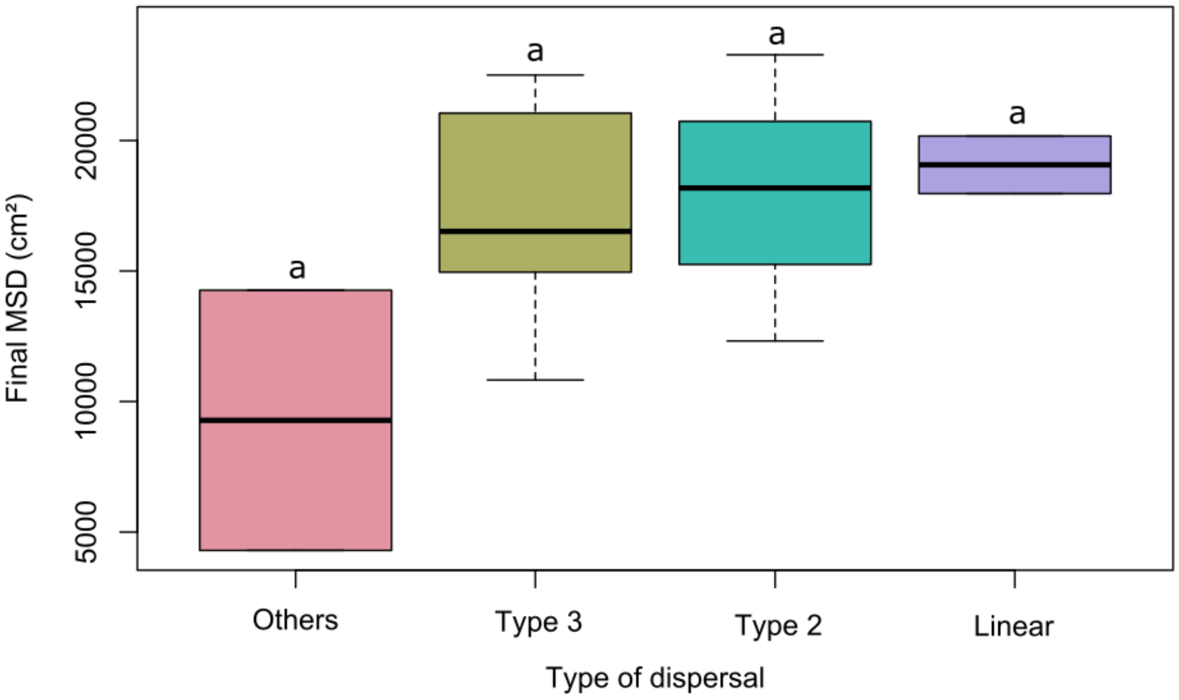

Figure S4. Boxplot of the final MSD according to the type of dispersal (anova; p = 0,19)

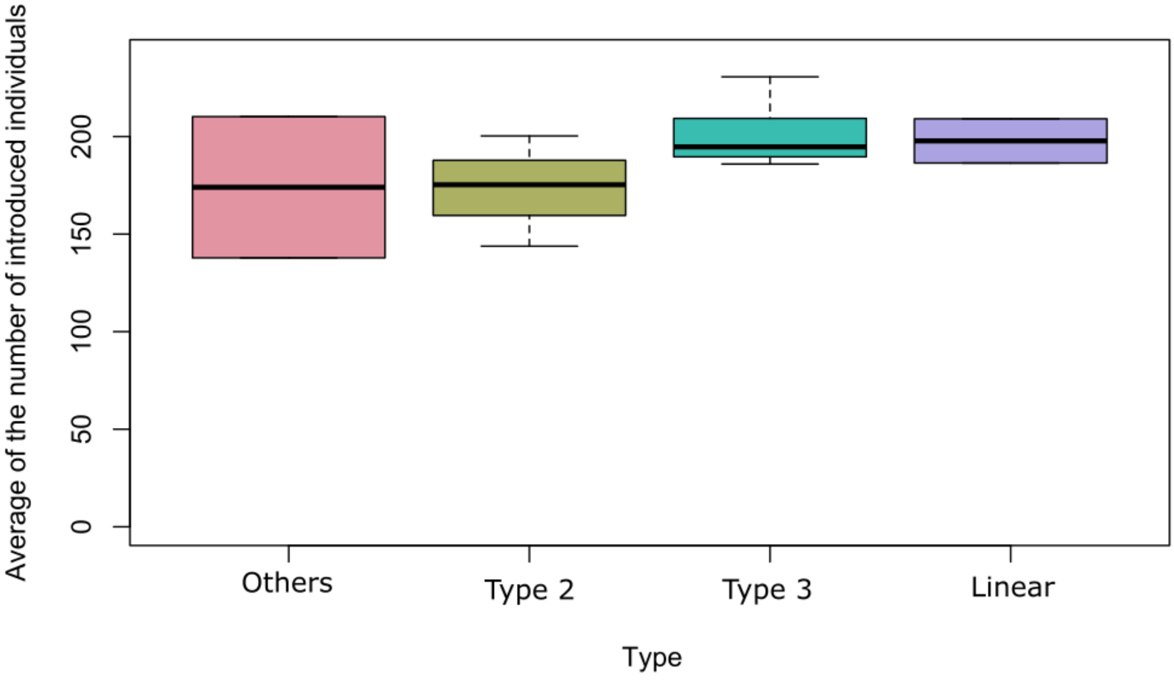

Figure S5. Boxplot of the average number of introduced individuals according to the type of dispersal. (anova; p=0.33)

Protocol P1

Quality of sealing

Materials

- A double spiral maze with a compartmentalized pathway in several zones (see Fig.S1)

- *Trichogramma* individuals ready to emerge

- Fresh and irradiated eggs of *Ephestia Khueniella* (surrogate host used for *Trichogramma* individuals rearing)

- A fine brush and a larger brush

- A preparation of Rubafix glue diluted with 2/3 water

The step-by-step protocol

1) Preparation of patches of eggs parasitized by *Trichogramma* individuals to be introduced in the areas for which sealing is to be tested.

The day before the emergence of *Trichogramma* individuals, cut a breeding strip in 6 or 7 pieces of 1cm by 0.5 cm. On average, if the eggs of *Ephestia Khueniella* have been deposited and that the parasitism has gone well (i.e. at least 80% of the deposited eggs have blackened), these cuts contain about 90 parasitized eggs.

2) Preparation of the maze

Take care of the cleaning of the maze, use ethanol to clean it and the two plexiglass plates which frame it.

First place the first Plexiglas plate, then a sheet of tracing paper cut to the right dimensions (those of the plates), then the spiral plate. The tracing paper must be carefully placed because it must be pierced by the clamping screws without creating an opening larger than the diameter of the screws in order not to compromise the proper conduct of the experiment. Then place the cuttings of the breeding strip in the different zones of the maze chosen. As the objective is to test for leaks, it is necessary to ensure that the areas to be tested are not too close to each other so that the identification of the possible leak is possible (no confusion possible as to the area where the leak comes from). In areas where no egg patches have been deposited, spread glue with the broad brush and deposit a few non-parasitized E. Khueniella eggs, with the fine brush, (between 10 and 30 depending on the size of the area, evenly distributed) in the area. These will serve as "sentinel" eggs. If there is no leakage, they should not blacken, if there is leakage, they should be (at least for some of them) parasitized and therefore blacken.

Once this last action is done, cover the two previous plates with the last Plexiglas plate. Screw it on. Leave the experimental device at 25°C until emergence the next day.

3) Beginning of the observations

The next day, recover the plate and leave it under a magnifying glass (exposed to the ambient temperature, in handling this one was of 20°C on average) in order to observe every 30min if possible, leaks did not take place. During these observations, it is possible that the leaking *Trichogramma* individuals are in fact hidden between a plexiglass plate and the spiral plate or that a leakage has occurred in the half hour of beating and that the trichogram has returned to the tested area. It is to compensate for what escapes the eye that sentinel eggs are used. But these regular observations are still useful because if an escape is detected, it will be possible to estimate the time from which it took place.

4) End of the test

After 48 hours, place the device in the cold (freezer) for about 10 minutes in order to immobilize the individuals still alive in the tested zones. Then recover the device, open it, clean the plates with water and ethanol. Before delicately removing the tracing paper on which the sentinel eggs are located, using a paper moistened with ethanol, recover the individuals immobilized by the cold in the tested zones. Once this is done, the tracing paper can be removed and set aside, under protection, for at least 5 days at 25°C, in order to see if the eggs blacken or not. The fact of having glued the eggs on the tracing paper and not having left them free, will allow a posteriori deduction of the possible areas of leakage.

After five days, recover the tracing paper and check the state of the eggs, if blackened, they have been parasitized.

Protocol P2

Introduction of individuals

Materials

- An introduction tube with individuals that emerged the day before

- A double spiral maze

The day before an experimental session with the double spiral maze device, the individuals to be used are isolated in a tube whose diameter is adapted to the diameter of the introduction hole (in our case so that the individuals are 24 hours old).

On the day of the test, the tube containing the individuals to be introduced is opened at one end. The open end is inserted into the insertion hole provided for this purpose. It is then necessary to tap the tube vigorously and repeatedly to drop the individuals into the device. According to the introduction tests carried out, the average rate of introduction of individuals is 65%. It is imperative to take this information into account in order to count enough individuals in the introduction tube to reach the population density to be studied in the device. Once the individuals have been introduced, the device is closed with a piece of glass that plugs the introduction hole.

The individuals introduced are then counted, as well as those remaining in the introduction tube, in order to deduce the introduction rate. In order to estimate the quantity of *E. Khueniella* eggs from which *Trichogramma* individuals are ready to emerge necessary to reach a satisfactory density of individuals, the parasitized (black) eggs are counted a posteriori under a binocular magnifying glass.
